# In situ nitrogen mineralization and nutrient release by soil amended with black soldier fly frass fertilizer

**DOI:** 10.1038/s41598-021-94269-3

**Published:** 2021-07-20

**Authors:** Dennis Beesigamukama, Benson Mochoge, Nicholas Korir, Changeh J. Ghemoh, Sevgan Subramanian, Chrysantus M. Tanga

**Affiliations:** 1 International Centre of Insect Physiology and Ecology, P. O. Box 30772-00100, Nairobi, Kenya; 2 Department of Agricultural Science and Technology, Kenyatta University, P.O. Box 43844-00100, Nairobi, Kenya; 3 Department of Crop Production and Management, Busitema University, P.O. Box 236, Tororo, Uganda; 4 Centre for African Bio-Entrepreneurship (CABE), Lavington, P.O. Box 25535-00603, Nairobi, Kenya

**Keywords:** Biochemistry, Ecology, Microbiology, Plant sciences, Environmental sciences

## Abstract

Although black soldier fly frass fertilizer (BSFFF) is effective on crop performance, information on nitrogen (N) mineralization and nutrient release capacity of soils amended with BSFFF is lacking. This study utilized field incubation experiments to investigate the ammonification, nitrification, microbial populations, and quantities of nutrients released by soils amended with BSFFF and commercial organic fertilizer (SAFI) for a period equivalent to two maize cropping seasons. For the control treatment, no BSFFF or SAFI was added. Results indicated that most of the N in BSFFF amended soils was available in the ammonium form, while soils treated with SAFI had higher nitrate concentration. The BSFFF amended soils experienced shorter net immobilization periods of N (30–60 days) compared to SAFI treated soils (60–95 days). Increased rates of mineralization (3–10 times) and nitrification (2–4 times) were observed in soils treated with BSFFF during the second season of application. The BSFFF treated soils showed significantly higher N, phosphorus, and magnesium release than the control. Repeated application of BSFFF led to increased N release by three-folds in the soil. Furthermore, soil amendment with BSFFF increased the populations of bacteria and fungi, reduced soil acidity, and increased phosphorus (two-folds) and magnesium (two–four-folds) release than SAFI treated soils. Our findings highlight the crucial role of BSFFF in improving soil health by addressing the challenges of soil acidity, phosphorus fixation and nutrient mining, which is characteristic of most tropical soils.

## Introduction

Crop production in sub-Saharan Africa (SSA) is hindered by low soil fertility^1–3^, yet mineral fertilizer use is limited by high costs and unavailability from a local source^4^. The use of organic fertilizers is still limited by their low availability due to other competing uses on the farm, such as feeding animals^5,6^ and domestic use as fuel^7^. Such competing uses leave little or none of the organic resources for use in crop production.

Insect frass is gaining global recognition as organic fertilizer for crop production and soil health improvement^8–13^. The black soldier fly (BSF) produces nutrient-rich frass fertilizer with minimal pathogens^10,14–16^ and chemical pollutants^17^. Unlike the conventional composting process, BSF-assisted composting is faster^18^ and more efficient at nutrient recycling^15,19^. In addition to its agronomic superiority^8,10,20,21^, the black soldier fly frass fertilizer (BSFFF) effectively improves plant health by suppressing plant pathogens^11,22^. These attributes make the BSFFF a quality organic fertilizer product worth integrating into farming systems of SSA, where sole mineral fertilizers are less effective due to multiple soil degradation challenges^23,24^.

The effectiveness of organic fertilizers in crop production is primarily influenced by the mineralization process, which determines the quantity and time in which nutrients are available for plant uptake^25,26^. Detailed information on the mineralization process of BSFFF amended soils is still limited. The only study on mineralization of BSFFF was done by Adin Yéton et al.^27^, who used a litter bag experiment that did not incorporate the frass into the soil, thereby ignoring the soil factors that influence nutrient release^28–30^. Furthermore, most studies on mineralization and nutrient release have focused on nitrogen only^25,31–34^, thus giving less attention to quantities of other macro and secondary nutrients released from organic fertilizers, which is important because organic farming systems rely on organic fertilizers to supply all the nutrients required by the plant throughout the growth cycle. The inadequate knowledge of nutrients’ release capacity other than N makes it difficult to solely rely on organic inputs, such as the BSFFF for nutrient supply.

Most plants consume nitrate-nitrogen (N); therefore, understanding the ammonification and nitrification process is crucial in designing strategies for increasing the agronomic use efficiency of organic fertilizers^35^. Although the mineralization of different organic fertilizers has been widely studied^26,31,36,37^, there is inadequate research on the ammonification and nitrification processes of soils amended with BSFFF. Yet, such information would guide optimal nutrient management strategies. For example, knowledge on length of N immobilization period and quantity of N immobilized could guide the amount of inorganic N supplementation required to satisfy plant N demands^21,31^.

The amount of nutrients released by organic fertilizers has been chiefly estimated using aerobic incubations under controlled conditions^34,38,39^. However, it is important to note that fluctuations highly influence the mineralization process in environmental conditions, such as temperature, rainfall, microbial abundance, and soil moisture content^35^. This, therefore, makes it difficult to transfer results from laboratory experiments to the field phase since incubation conditions make the mineralization process optimal. The buried-bag technique is one of the popular methods for studying mineralization because it is easy, causes minimal disturbance of the soil, and allows investigation of nutrient dynamics of subsurface soil layers, and is thus suitable for agronomic studies^36^. Therefore, this study aimed to determine the rates of N mineralization and nitrification and the amount of macro and secondary nutrients released by soil amended with BSFFF under open field conditions to generate the information necessary for the recommendation of BSFFF into existing farming practices.

## Results

### Ammonium and nitrate concentrations, and ammonium/nitrate ratio

The concentrations of ammonium in soil varied significantly due to fertilizer amendments (short rainy season: χ^2^ = 123.7, df = 2, p < 0.001, long rainy season: χ^2^ = 129.2, df = 2, p < 0.001), incubation time (short rainy season: χ^2^ = 69.6, df = 5, p < 0.001, long rainy season: χ^2^ = 34.3, df = 5, p < 0.001). The interaction effect of fertilizer amendments and incubation time was also significant (short rainy season: χ^2^ = 52.4, df = 10, p < 0.001, long rainy season: χ^2^ = 30.3, df = 5, p < 0.001). Initial ammonium concentrations ranged between 1.1 and 402 mg kg^−1^, whereby the control soil and BSFFF treated soil had the lowest and highest ammonium concentrations, respectively (Table 1). The ammonium concentrations of BSFFF and commercial organic fertilizer (SAFI) treated soils were 2–287 and 4–122 times higher than those of the unamended soil, respectively. The ammonium concentration of soil amended with BSFFF and SAFI followed a decreasing trend throughout experiments. On the other hand, the ammonium concentration of unamended soil was observed to increase considerably to reach its peak values at the 60th day of incubation.

**Table 1:** Concentrations of ammonium and nitrates, and ammonium-nitrate ratio of soil amended with BSF frass and commercial organic fertilizers.

Parameter	Time (days)	Season 2019A (Short rains)				Season 2019B (Long rains)			
		Control	BSFFF	SAFI	p value	Control	BSFFF	SAFI	p value
Ammonium (mg kg^−1^)	0	1.1 ± 0.3c	300.6 ± 34.6a	128.2 ± 10.6b	***	1.4 ± 0.3c	402.2 ± 46.4a	171.5 ± 14.1b	***
	15	2.2 ± 0.3b	207.1 ± 75.3a	96.3 ± 5.7ab	*	3.8 ± 0.5b	353.6 ± 128.3a	164.3 ± 9.9ab	*
	30	0.8 ± 0.0b	119.9 ± 27.6a	34.0 ± 5.6b	**	2.4 ± 0.0b	359.7 ± 82.9a	79.3 ± 7.5b	**
	60	50.7 ± 18ab	128.9 ± 29.5a	6.0 ± 4.2b	*	220.3 ± 48.1ab	490.7 ± 112.5a	22.1 ± 15.6ab	*
	90	0.8 ± 0.0b	51.3 ± 16.6a	3.4 ± 1.5b	*	2.1 ± 0.1b	145.2 ± 46.9a	9.5 ± 4.3b	*
	125	2.1 ± 0.7b	71.9 ± 17.6a	1.8 ± 0.6b	***	6.4 ± 2.0b	215.7 ± 52.7a	5.5 ± 1.8b	***
Nitrate (mg kg^−1^)	0	0.2 ± 0.0b	3.9 ± 1.3b	208.1 ± 36.3a	***	0.4 ± 0.1b	7.5 ± 2.5b	400.2 ± 69.8a	***
	15	2.9 ± 0.3b	1.2 ± 0.4b	230.3 ± 6.7a	***	5.5 ± 0.7b	2.4 ± 0.7b	444.8 ± 12.9a	***
	30	3.2 ± 0.5b	2.1 ± 0.4b	130.7 ± 11.8a	***	9.1 ± 1.4b	6.1 ± 1.3b	377.8 ± 34.0a	***
	60	9.5 ± 2.1	6.5 ± 1.9	91.5 ± 37.1	ns	14.1 ± 3.1	9.7 ± 2.8	136.0 ± 55.1	ns
	90	13.9 ± 1.9b	5.3 ± 1.9b	234.1 ± 47.7a	**	18.1 ± 2.5b	6.9 ± 2.5b	305.2 ± 62.1a	**
	125	8.4 ± 2.0b	9.1 ± 0.5b	170.2 ± 60.3a	*	14.8 ± 3.5b	16.0 ± 0.9b	298.8 ± 105.8a	*
Ammonium/Nitrate ratio	0	5.7 ± 1.4a	90.0 ± 21.3a	0.66 ± 0.1b	**	4.0 ± 1.0b	62.6 ± 14.8a	0.5 ± 0.1b	**
	15	0.8 ± 0.1a	165.1 ± 10.6a	0.42 ± 0.0b	***	0.70 ± 0.1b	145.9 ± 9.3a	0.4 ± 0.0b	***
	30	0.3 ± 0.0b	57.3 ± 3.6a	0.27 ± 0.1b	***	0.3 ± 0.1b	59.4 ± 3.8a	0.2 ± 0.0b	***
	60	5.1 ± 1.1ab	22.1 ± 7.2a	0.27 ± 0.3b	*	15.6 ± 0.2ab	56.5 ± 18.5a	0.7 ± 0.6b	*
	90	0.1 ± 0.0b	13.1 ± 5.2a	0.01 ± 0.0b	*	0.1 ± 0.0b	28.6 ± 11.3a	0.03 ± 0.0b	*
	125	0.3 ± 0.1b	8.1 ± 2.2a	0.01 ± 0.0b	**	0.5 ± 0.2b	13.8 ± 3.8a	0.02 ± 0.0b	**

*** p < 0.001, **p < 0.01, *p < 0.05, ns = not significant at p ≥ 0.05, BSFFF = Black soldier fly frass fertilizer, SAFI = commercial organic fertilizer, control = unamended soil. Within the same row and per parameter, means (± standard error) with the same letters are not significantly different at p ≥ 0.05, n = 3.

There were significant decreases in the ammonium concentration of BSFFF treated soil from initial values up to the 60th day of incubation during the short rainy season (p < 0.001) and from the 60th to 90th day after incubation during the long rainy season (p < 0.001). Soil amended with BSFFF had significantly higher ammonium concentration than the control treatment throughout experiments, except at 60 days after incubation during both seasons. Likewise, soil treated with BSFFF achieved significantly higher ammonium concentration than SAFI treatments, except at 15 and 60 days after incubation during the short and long rainy season experiments, respectively. At the end of experiments, soil amended with BSFFF had the highest ammonium concentration, which was 34 and 40 times higher than those of the control and SAFI treatments, respectively (Table 1).

Significant differences in soil nitrate concentration due to fertilizer treatments (short rainy season: χ^2^ = 242.0 df = 2, p < 0.001, long rainy season: χ^2^ = 299.0, df = 2, p < 0.001) and the interaction of treatment and incubation time (short rainy season: χ^2^ = 23.2, df = 10, p = 0.01, long rainy season: χ^2^ = 31.3, df = 10, p < 0.001) were observed. The effect of incubation time was significant during the long rainy season only (short rainy season: χ^2^ = 11.0, df = 5, p = 0.05, long rainy season: χ^2^ = 13.0, df = 5, p = 0.02). Initial nitrate concentrations ranged between 0.2 and 400 mg kg^−1^, while the soil in the control treatment and that treated with SAFI had the lowest and highest values, respectively (Table 1). The nitrate concentration of the control treatment increased to peak values (14–18 mg kg^−1^) at the 90th day after incubation during both seasons and decreased afterwards.

The nitrate concentrations of soil amended with BSFFF and SAFI did not follow a uniform trend. Soil treated with BSFFF reached peak nitrate concentration 125 days after incubation, while SAFI amended soil achieved the highest nitrate values after 90 and 15 days of incubation during the short and long rainy seasons, respectively. The nitrate concentration of soil amended with SAFI fertilizer significantly (p = 0.02) decreased from the 30th to 60th day after incubation during the long rainy season. The peak nitrate concentration of SAFI treated soil was 17–27 and 26–28 times higher than those achieved using the control and BSFFF treatments, respectively. Soil amended with SAFI fertilizer maintained significantly higher nitrate concentrations than the control and BSFFF treatments, except at 60 days after incubation during both seasons. At 125 days after incubation, the nitrate concentration soil amended with SAFI was 20 and 19 times higher than those of the control and BSFFF treatments, respectively (Table 1).

The ammonium/nitrate ratio was significantly influenced by fertilizer treatments (short rainy season: χ^2^ = 366.9, df = 2, p < 0.001, long rainy season: χ^2^ = 316.4, df = 2, p < 0.001), incubation time (short rainy season: χ^2^ = 167.6, df = 5, p < 0.001, long rainy season: χ^2^ = 80.2, df = 5, p < 0.001) and their interactions (short rainy season: χ^2^ = 329.3, df = 10, p < 0.001, long rainy season: χ^2^ = 160.4, df = 10, p < 0.001). The initial ammonium/nitrate ratio ranged between 0.5 and 90, while SAFI and BSFFF amended soils had the lowest and highest values, respectively (Table 1). There was a spike in ammonium/nitrate ratios of the control soil to peak values at 60 days after incubation during both seasons. The same ammonium/nitrate ratio was observed in soil amended with SAFI during the long rainy season. The ammonium/nitrate ratio of soil amended with BSFFF significantly (p < 0.001) increased to peak values at 15 after incubation and decreased significantly up to 60 days of incubation. Thereafter, the ammonium/nitrate ratio decreased until the end of experiments.

Soils amended with SAFI maintained a significantly lower ammonium/nitrate ratio than BSFFF treated soils (Table 1). The BSFFF amendment caused significantly higher ratios of ammonium/nitrate than the control treatment at 30 days (F = 247.3, df = 2, 6, p < 0.001), 90 days (F = 6.4, df = 2, 6, p = 0.03) and 125 days (F = 12.8, df = 2, 6, p < 0.001) after incubation during the short rainy season. Furthermore, BSFFF amended soil had significantly higher ammonium/nitrate ratio than the control during long rainy season, except at 60 days after incubation (F = 7.3, df = 2, 6, p = 0.03) (Table 1). At the end of experiments, the ammonium ratios to nitrate ranged between 0.01 and 13.8, while SAFI and BSFFF amended soils had the lowest and highest values, respectively.

### Mineralization and nitrification rates

The soil nitrogen mineralization rate was significantly influenced by incubation time (short rainy season: χ^2^ = 25.7, df = 4, p < 0.001, long rainy season: χ^2^ = 11.0, df = 4, p = 0.027) and the interaction of incubation time and fertilizer amendment (short rainy season: χ^2^ = 34.1, df = 8, p < 0.001, long rainy season: χ^2^ = 45.0, df = 8, p < 0.001). The effect of fertilizer amendment was significant during the short rainy season only (χ^2^ = 6.1, df = 2, p = 0.046). The fluxes of nitrogen mineralization of soil amended using different treatments are presented in Table 2.

**Table 2:** Nitrogen mineralization and nitrification rates of soil amended with BSF frass and commercial organic fertilizers.

Parameter	Time (days)	Season 2019A (Short rains)				Season 2019B (Long rains)			
		Control	BSFFF	SAFI	p value	Control	BSFFF	SAFI	p value
Mineralization rate (mg N kg^−1^ day^−1^)	15	0.25 ± 0.02	− 3.18 ± 0.07	− 0.65 ± 2.34	ns	0.50 ± 0.07	3.08 ± 4.53	2.49 ± 4.11	ns
	30	− 0.08 ± 0.03a	− 5.76 ± 0.03ab	− 10.79 ± 1.11b	*	0.15 ± 0.12	0.65 ± 5.04	− 10.14 ± 3.52	ns
	60	1.88 ± 0.06	0.45 ± 0.07	− 2.24 ± 1.57	ns	7.43 ± 1.68a	4.49 ± 2.77a	− 9.97 ± 2.63b	**
	90	− 1.52 ± 0.01b	− 2.62 ± 0.06b	4.67 ± 2.14a	*	− 7.14 ± 1.62b	− 11.61 ± 3.29b	5.22 ± 2.66a	*
	125	− 0.12 ± 0.07	0.70 ± 0.05	− 1.87 ± 1.65	ns	0.03 ± 0.16	2.27 ± 2.73	− 0.297 ± 2.79	ns
Nitrification rate (mg NO_3_^–1^ kg^−1^ day^−1^)	15	0.18 ± 0.04	− 0.18 ± 3.01	1.48 ± 1.99	ns	0.34 ± 0.04	− 0.35 ± 0.13	2.97 ± 4.50	ns
	30	0.02 ± 0.05a	0.06 ± 3.64a	− 6.64 ± 1.15b	***	0.24 ± 0.09	0.25 ± 0.07	− 4.47 ± 2.87	ns
	60	0.21 ± 0.66	0.15 ± 0.81	− 1.31 ± 1.34	ns	0.17 ± 0.08a	0.12 ± 0.10a	− 8.06 ± 2.85b	*
	90	0.15 ± 0.61	− 0.04 ± 0.89	4.76 ± 2.08	ns	0.13 ± 0.02	− 0.09 ± 0.08	5.64 ± 2.95	ns
	125	− 0.16 ± 0.09	0.11 ± 0.95	− 1.83 ± 1.67	ns	− 0.09 ± 0.11	0.25 ± 0.06	− 0.18 ± 2.67	ns

***p < 0.001, **p < 0.01, *p < 0.05, ns = not significant at p ≥ 0.05, BSFFF = Black soldier fly frass fertilizer, SAFI = commercial organic fertilizer, control = unamended soil. Within the same row and per parameter, means (± standard error) followed by the same letters are not significantly different at p ≥ 0.05, n = 3.

Amendment with BSFFF triggered net mineralization rates at 60 and 125 days after incubation during the short rainy season and most of the long rainy season, except at 90 days after incubation. Soil amended with SAFI achieved net mineralization in 90 days after incubation during both seasons and at 15 days after incubation during the long rainy season. For the unamended soil, net mineralization rates were recorded at 15 and 60 days after incubation during the short rainy season, and most of the long rainy season, except at 90 days after incubation (Table 2).

Soil amended with SAFI experienced longer periods (60–95 days) of N immobilization during both seasons than BSFFF treatments (30–60 days) (Table 2). During the short rainy season, the highest immobilization rate (− 11 mg N kg^−1^ day^−1^) was recorded at 30 days after incubation in soil amended with SAFI, and this was significantly (p < 0.038) higher than that of BSFFF and control treatments. Soil amended with BSFFF experienced the highest immobilization rate (− 12 mg N kg^−1^ day^−1^) during the long rainy season, which was not significantly different (p < 0.01) from that of the untreated soil.

All treatments achieved peak N mineralization rates (0.5–5.2 mg N kg^−1^ day^−1^) between 60 and 90 days after incubation (Table 2). Soil amended using SAFI achieved the highest net mineralization rate during the short rainy season at 90 days after incubation. During the long rainy season, the unamended soil had the highest mineralization rate (7.4 mg N kg^−1^ day^−1^) 60 days after incubation, which was not significantly different from that of BSFFF treatment. Generally, all treatments experienced rises in the rates of N mineralization during the long rainy seasons, with higher increases (three–ten folds) observed in BSFFF treated soils.

The soil nitrification rate was significantly influenced by incubation time (short rainy season: χ^2^ = 21.6, df = 4, p < 0.001, long rainy season: χ^2^ = 10.9, df = 4, p = 0.03) and the interaction of incubation time and fertilizer treatments (short rainy season: χ^2^ = 43.2, df = 8, p < 0.001, long rainy season: χ^2^ = 24, df = 8, p = 0.002). Table 2 presents the changes in nitrification rates during experiments. All treatments reached peak net nitrification rates 60–90 days after incubation. Soil amended with SAFI had the highest net nitrification rates (1.5–5.6 mg N kg^−1^ day^−1^), which were 22–25 and 17–75 times higher than the values achieved using BSFFF and control treatments, respectively. However, amendment with SAFI depressed the nitrification rate (− 0.18 to − 8.1 mg N kg^−1^ day^−1^) more than BSFFF treatments (− 0.04 to − 0.35 mg N kg^−1^ day^−1^).

Soil amended with BSFFF achieved a longer period of net nitrification (80 days) than SAFI amended soil (45 days) (Table 2). The net nitrification rates of soils amended with BSFFF were significantly higher than those achieved using SAFI at 30 and 60 days after incubation during the short (p < 0.001) and long rainy seasons (p = 0.019), respectively. The net nitrification rates of BSFFF amended soils increased by two–four folds during the long rainy season.

### Soil pH, and populations of bacteria and fungi

**Figure 1:**
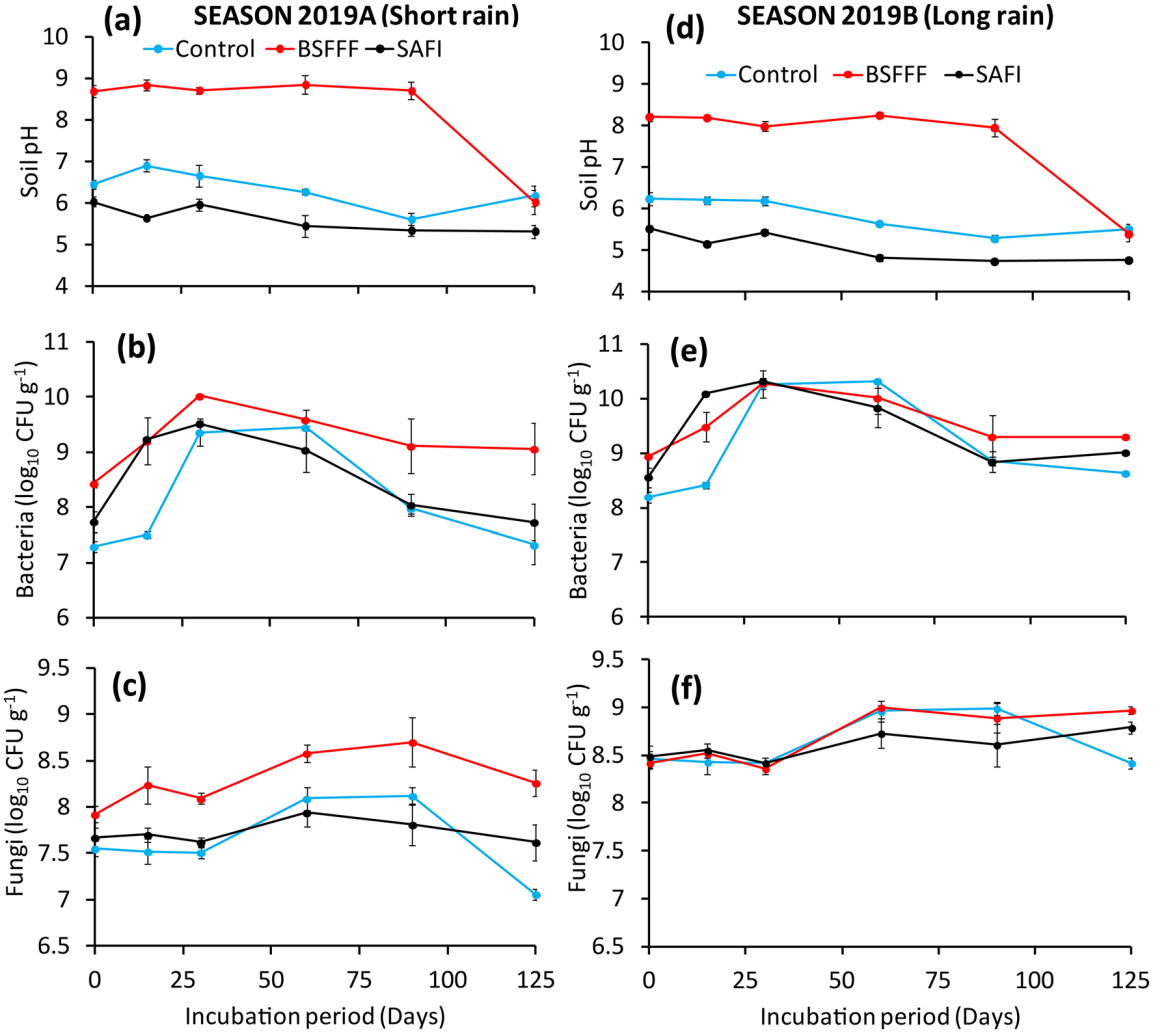
Changes in pH (**a**,**d**), and populations of bacteria (**b**,**e**) and fungi (**c**,**f**) of soil treated with different fertilizers during the short (**a**–**c**) and long rainy (**d**–**f**) seasons incubation experiments.

The soil pH was significantly influenced by fertilizer treatments (short rainy season: χ^2^ = 733.7, df = 2, p < 0.001, long rainy season: χ^2^ = 192.8, df = 2, p < 0.001), incubation time (short rains season: χ^2^ = 119.2, df = 5, p < 0.001, long rainy season: χ^2^ = 387.2, df = 5, p < 0.001) and their interaction (short rainy season: χ^2^ = 127.1, df = 10, p < 0.001, long rainy season: χ^2^ = 315.4, df = 10, p < 0.001). The initial pH of soils amended with different fertilizer treatments ranged between 5.2 and 8.7. Thereafter, the soil pH decreased until the end of the experiments (Fig. 1a,d). Soil amended with BSFFF maintained significantly (p < 0.001) higher pH values than control soil up to 90 days of incubation and throughout experiments for SAFI treated soil. The unamended soil had significantly higher pH than that of soil treated with SAFI, except at 90 days after incubation during the short rainy season (Fig. 1a). At the end of experiments, soil pH ranged between 4.8 and 6.2, whereby the control soil had the highest pH while soil amended with SAFI had the lowest pH.

The soil bacteria population was significantly influenced by incubation time during both seasons (short rainy season: χ^2^ = 24.7, df = 5, p < 0.001, long rainy season: χ^2^ = 73.9, df = 5, p < 0.001) and fertilizer amendment during the short rainy season only (χ^2^ = 19.9, df = 2, p < 0.001). However, the interaction of incubation time and fertilizer amendments was not significant (short rainy season: χ^2^ = 8.9, df = 10, p = 0.541, long rainy season: χ^2^ = 8.1, df = 10, p = 0.618). The bacterial population significantly increased from initial values to peak levels (9.5–10.3 log_10_ CFU g^−1^) between 30 and 60 days after incubation and decreased afterwards (Fig. 1b,e). Soil treated with BSFFF achieved the highest bacterial populations during both seasons. Its peak bacterial population during the long rainy season was equivalent to the highest value obtained using SAFI (Fig. 1e). Amendment with BSFFF caused significantly higher bacterial populations than other treatments at 30 and 90 days during the short rainy season and at 125 days after incubation during both seasons. At the end of experiments, the bacterial populations ranged between 7.3 and 9.3 log_10_ CFU g^−1^, whereby BSFFF and control treatments had the highest and lowest values, respectively.

The different fertilizer amendments caused significant differences in soil fungi populations during the short rainy season only (χ^2^ = 15.5, df = 2, p < 0.001). The effect of incubation time was significant during both seasons (short rainy season: χ^2^ = 11.9, df = 5, p = 0.04, long rainy season: χ^2^ = 53.7, df = 5, p < 0.001) while the interaction of fertilizer treatments and incubation time was significant during the long rainy season only (χ^2^ = 18.7, df = 10, p = 0.044). Minimal changes in fungi populations were observed in the first 30 days of incubation (Fig. 1c,f). The soil fungi populations significantly increased to peak values (7.9–9.0 log_10_ CFU g^−1^) between 60 and 90 days after incubation and decreased afterwards. However, SAFI treated soil achieved peak fungi populations after 125 days during the long rainy season. Soil amended with BSFFF maintained significantly (p < 0.001) higher fungi populations than other treatments during the entire short rainy season and 125 days after incubation during the long rainy season. At the end of the experiments, the control treatment had the lowest fungi population (7.1 log_10_ CFU g^−1^) while BSFFF treated soil had the highest (9.0 log_10_ CFU g^−1^). Also, soils treated with BSFFF and SAFI had a significantly (p < 0.001) higher fungi population than the unamended soil at the end of the experiments.

### Soil nutrients concentration

**Figure 2:**
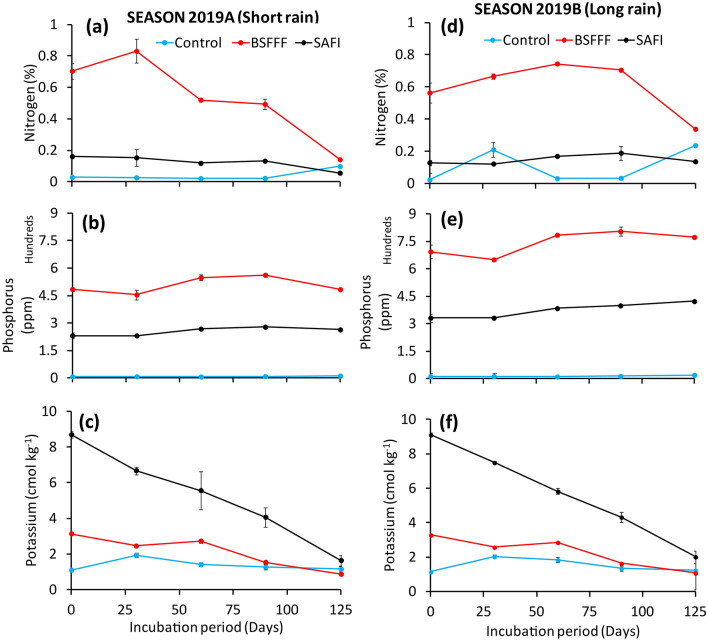
Effects of different fertilizers on concentrations of total nitrogen (**a**,**d**), available phosphorus (**b**,**e**) and exchangeable potassium (**c**,**f**) of soils during the short (**a**–**c**) and long rainy (**d**–**f**) seasons incubation experiments.

The total N concentration was significantly influenced by fertilizer amendments (short rainy season: χ^2^ = 1011.7, df = 2, p < 0.001, long rainy season: χ^2^ = 1509.5, df = 2, p < 0.001), incubation time (short rainy season: χ^2^ = 143.1, df = 4, p < 0.001, long rainy season: χ^2^ = 30.2, df = 4, p < 0.001) and their interaction (short rainy season: χ^2^ = 261.9, df = 8, p < 0.001, long rainy season: χ^2^ = 231.6, df = 8, p < 0.001). The total N concentration of soil treated with BSFFF significantly increased to peak values (0.74–0.83%) at 30 and 60 days after incubation during the short and long rainy season, respectively, and decreased afterwards (Fig. 2a,d). On the other hand, there was minimal changes in the total N concentration in soil amended with SAFI during experiments, while total N concentration in unamended soil was observed to increase from day 60 after incubation and peaked on the 125th day.

Soils amended with BSFFF maintained significantly (p < 0.001) higher total N concentration than other treatments throughout experiments. The total N concentration of soil treated with BSFFF was 2.5–5.4 and 2.5–4.4 times higher than soil amended with SAFI during the short and long rainy seasons, respectively. Likewise, soil amended with SAFI achieved significantly (p < 0.001) higher total N concentration than the unamended soil, except at 30 days during the long rainy season and on the 125th day during both seasons. At the end of the experiments, soils treated with BSFFF had the highest N concentration (0.14–0.34%), which was significantly (p < 0.001) higher than that of the SAFI and control treatments by 149–152% and 32–44%, respectively.

The concentration of available phosphorus (P) was found to vary significantly due to fertilizer amendments (short rainy season: χ^2^ = 5920.2, df = 2, p < 0.001, long rainy season: χ^2^ = 6128.9, df = 2, p < 0.001), incubation time (short rainy season: χ^2^ = 55.1, df = 4, p < 0.001, long rainy season: short rainy season: χ^2^ = 68.0, df = 4, p < 0.001) and the interaction effect of fertilizers and incubation time (short rainy season: short rainy season: χ^2^ = 45.4, df = 8, p < 0.001, long rainy season: short rainy season: χ^2^ = 43.3, df = 8, p < 0.001). Initial P concentration ranged between 8 and 694 ppm and significantly (p < 0.001) increased to peak values (279–804 ppm) between 90 and 125 days after incubation (Fig. 2b,e).

All fertilizer treatments maintained significantly (p < 0.001) higher P levels than the control treatment throughout experiments. The P concentration of the control soil did not vary significantly during experiments. The P concentration of soil treated with BSFFF was significantly (p < 0.001) higher than that of SAFI treatment by 1.8–2.1 times. At the end of experiments, the P concentration of soil treated with BSFFF was about 83% higher than that of SAFI treatment (p < 0.001).

The concentrations of exchangeable potassium (K) also varied significantly due to fertilizer amendments (short rainy season: χ^2^ = 384.8, df = 2, p < 0.001, long rainy season: χ^2^ = 386.7, df = 2, p < 0.001), incubation time (short rainy season: χ^2^ = 154.7, df = 4, p < 0.001, long rainy season: χ^2^ = 140.9, df = 4, p < 0.001) and their interaction (short rainy season: χ^2^ = 127.6, df = 8, p < 0.001, long rainy season: χ^2^ = 118.7, df = 8, p < 0.001). The initial K concentration ranged between 1.1 and 9.1 cmol kg^−1^ from where it decreased throughout experiments, with the highest decreases observed in SAFI treated soils (Fig. 2c,f). Soil amended with SAFI maintained significantly (p < 0.001) higher K levels than other treatments, except at 125 days during the long rainy season. Soil treated with BSFFF had significantly (p < 0.001) higher K concentration than the control soil up to 90 days of incubation. The final K concentration of SAFI treated soil (1.6–2.0 cmol kg^−1^) was 87–89% and 42–63% higher than those achieved using BSFFF and the control treatment, respectively.

The exchangeable calcium (Ca) ions varied significantly due to fertilizer amendments during experiments (short rainy season: χ^2^ = 59.9, df = 2, p < 0.001, long rainy season: χ^2^ = 48.9, df = 2, p < 0.001) and incubation time (short rainy season: χ^2^ = 19.9, df = 4, p < 0.001, long rainy season: χ^2^ = 92.2, df = 4, p < 0.001) but the interaction effects were not significant (short rainy season: χ^2^ = 6.3, df = 8, p = 0.61, long rainy season: χ^2^ = 14.0, df = 8, p = 0.08). The Ca concentration decreased to minimum values at 90 and 60 days of incubation during the short and long rainy seasons, respectively and increased thereafter (Table 3). Soil amended with SAFI achieved significantly (p < 0.001) higher Ca concentration than BSFFF treatments by 24–96 and 26–91% during short and long rainy seasons, respectively.

**Table 3:** Concentrations of exchangeable calcium and magnesium in soil alone and soil amended by BSF frass and commercial organic fertilizers during the short and long rainy seasons.

Parameter	Time (days)	Season 2019A (Short rains)				Season 2019B (Long rains)			
		Control	BSFFF	SAFI	p value	Control	BSFFF	SAFI	p value
Calcium (cmol kg^−1^)	0	1.10 ± 0.04a	0.84 ± 0.02b	1.04 ± 0.04a	**	0.76 ± 0.03a	0.58 ± 0.01b	0.73 ± 0.03a	**
	30	1.08 ± 0.03a	0.82 ± 0.01b	1.11 ± 0.03a	***	0.75 ± 0.02a	0.56 ± 0.00b	0.77 ± 0.02a	***
	60	0.96 ± 0.02ab	0.71 ± 0.02b	1.04 ± 0.13a	ns	0.66 ± 0.02ab	0.49 ± 0.01b	0.73 ± 0.09a	*
	90	0.81 ± 0.01ab	0.53 ± .006b	1.02 ± 0.06a	*	1.05 ± 0.01ab	0.69 ± 0.07b	1.32 ± 0.20a	*
	125	0.97 ± 0.02	0.80 ± 0.01	1.16 ± 0.16	ns	1.06 ± 0.02	0.87 ± 0.01	1.26 ± 0.18	ns
Magnesium (cmol kg^−1^)	0	0.78 ± 0.04b	2.86 ± 0.11a	1.06 ± 0.02b	***	0.65 ± 0.03b	2.37 ± 0.09a	0.89 ± 0.02b	***
	30	0.83 ± 0.02c	2.84 ± 0.01a	1.09 ± 0.06b	***	0.70 ± 0.01c	2.36 ± 0.01a	0.91 ± 0.05b	***
	60	0.81 ± 0.10b	3.10 ± 0.21a	0.93 ± 0.02b	***	0.68 ± 0.04b	2.57 ± 0.08a	0.78 ± 0.18b	***
	90	0.71 ± 0.02b	2.56 ± 0.12a	0.89 ± 0.14b	***	0.58 ± 0.01b	2.11 ± 0.10a	0.74 ± 0.14b	***
	125	0.68 ± 0.02b	2.85 ± 0.03a	0.78 ± 0.19b	***	0.55 ± 0.02a	2.35 ± 0.02a	0.64 ± 0.15b	***

***p < 0.001, **p < 0.01, *p < 0.05, ns = not significant at p ≥ 0.05 BSFFF = Black soldier fly frass fertilizer, SAFI = commercial organic fertilizer, control = unamended soil. Within the same row and per parameter, means (± standard error) followed by the same letters are not significantly different at p ≥ 0.05, n = 3.

The control treatment attained significantly (p < 0.001) higher Ca concentration than BSFFF amended soil during the first 30 days of incubation. At the end of experiments, the Ca concentration did not vary significantly (long rainy season: p = 0.096, long rains season: p = 0.098), but SAFI amended soils had the highest. At the same time, BSFFF treated soil had the lowest Ca concentration.

The exchangeable magnesium (Mg) ions in the soil also varied significantly due to fertilizer amendments (short rainy season: χ^2^ = 1341.0 df = 2, p < 0.001, long rainy season: χ^2^ = 1339.4, df = 2, p < 0.001) and incubation time (short rainy season: χ^2^ = 12.0, df = 4, p = 0.018, long rains season: χ^2^ = 14.7, df = 4, p = 0.005) during both seasons, but their interactions were not significant (short rainy season: χ^2^ = 10.8, df = 8, p = 0.213, long rainy season: χ^2^ = 10.9, df = 4, p = 0.209). Initial Mg concentrations ranged between 0.78 and 2.86 cmol kg^−1^, while the control and BSFFF treatments had the lowest and highest Mg concentrations, respectively (Table 3).

The Mg concentration increased to peak values of 0.68 to 3.10 cmol kg^−1^ between 30 and 60 days of incubation, after which the concentrations kept decreasing up to the end of the experiments. The Mg concentration of soil amended with BSFFF was 2.6–3.7 and 3.4–4.3 times higher than those of SAFI treated and unamended soil, respectively. Also, soils treated with BSFFF maintained significantly (p < 0.001) higher Mg concentration than other treatments, except the control at 125 days of incubation during the long rainy season. At the end of the experiments, soil treated with BSFFF had the highest Mg concentration (2.4–2.9 cmol kg^−1^), which was 3.7 and 4.3 times higher than those of SAFI and control treatments.

### Nutrients released by unamended soil and soils amended with organic fertilizers

The amount of N (short rainy season: F = 5.5, df = 2, 6, p = 0.044, long rainy season: F = 6.2, df = 2, 6, p = 0.035), that of available P (short rainy season: F = 740.9, df = 2, 6, p < 0.001, long rainy season: F = 740.2, df = 2, 6, p < 0.0001) and exchangeable Mg (short rainy season: F = 135.7, df = 2, 6, p < 0.001, long rainy season: F = 28.6, df = 2, 6, p < 0.001) released by the unamended soil and soils amended with different organic fertilizers varied significantly during the experiments (Table 4). However, the quantities of K (short rainy season: F = 3.4, df = 2, 6, p = 0.102, long rainy season: F = 4.0, df = 2, 6, p = 0.078) and Ca (short rainy season: F = 4.2, df = 2, 6, p = 0.073, long rainy season: F = 4.1, df = 2, 6, p = 0.074) released were not significantly influenced by fertilizer amendments.

**Table 4:** Nutrients released from unamended soil and soils amended with organic fertilizers during the short and long rains cropping seasons.

Treatment	Season 2019A (Short rains)					Season 2019B (Long rains)				
	Mineral nitrogen	Available phosphorus	Exchangeable cations			Mineral nitrogen	Available phosphorus	Exchangeable cations		
			Potassium	Calcium	Magnesium			Potassium	Calcium	Magnesium
	(kg ha^−1^)									
Control	27.9 ± 6.1b	32.1 ± 6.5c	1197.4 ± 173.1	516.7 ± 19.3	215.5 ± 10.5b	56.1 ± 12.0b	51.0 ± 10.4c	1277.1 ± 184.4	561.6 ± 20.9	174.6 ± 8.7c
BSFFF	215.2 ± 9.0ab	1276.2 ± 38.6b	899.6 ± 101.2	423.1 ± 5.6	902.2 ± 16.7a	616.0 ± 147.7a	2039.8 ± 61.7a	1111.0 ± 125.3	460.1 ± 6.1	743.9 ± 13.8a
SAFI	446.4 ± 146.7a	696.2 ± 6.2a	1692.3 ± 316.7	608.3 ± 76.0	245.2 ± 52.3b	389.2 ± 127.8ab	1112.6 ± 9.9b	2081.8 ± 389.1	660.6 ± 82.4	408.5 ± 91.3b
p value	*	***	ns	Ns	***	*	***	ns	ns	***

***p < 0.001, **p < 0.01, *p < 0.05, ns = nonsignificant at p ≥ 0.05, BSFFF = Black soldier fly frass fertilizer, SAFI = commercial organic fertilizer, control = unamended soil. Within the same column and per parameter, means (± standard error) followed by the same letters are not significantly different at p ≥ 0.05, n = 3.

Soil amended with SAFI and BSFFF released significantly (p < 0.001) higher N than unamended soil during the short and long rainy seasons, respectively (Table 4). The N released by soil treated with SAFI and BSFFF was 7–16 times and 8–11 times higher than N released by the control soil, respectively. Soils treated with SAFI released 107% higher N than BSFFF treated soil during the short rainy season. On the other hand, soil treated with BSFFF released the highest N during the long rainy season, 58 and 998% higher than SAFI amended soil and the control soil, respectively.

The quantity of P released by BSFFF amended soils was significantly (p < 0.001) higher than those of SAFI and control treatments by about two- and forty-folds, respectively. In contrast, the P released by soil treated with SAFI was 22 times higher than that released by the control soil (Table 4). Soil treated with SAFI had the highest K, which was 1.9 times and 1.4–1.6 times higher than those of BSFFF and control treatments, respectively. The Ca released by soil amended with SAFI was 44 and 18% higher than that released by BSFFF and the control treatments, respectively.

The highest quantity of Mg was released by soil amended with BSFFF, and this was significantly (p < 0.001) higher than those of SAFI and control treatments by 82–268% and 319–326%, respectively (Table 4). However, soil amendment with SAFI released significantly higher Mg than the control treatment.

## Discussion

The patterns of soil mineralization and nitrification rates, and seasonal differences observed during this study (Tables 1 and 2) have been previously reported^35,40^. The high ammonium and low nitrate concentrations observed in BSFFF treated soil could be attributed to the quality of the frass fertilizer used in the study (Table 5). Ammonium nitrogen is one of the major N fractions that influence N released from organic fertilizers^32^. The BSF frass contains high levels of ammonium nitrogen^15,18,41^, which requires time to be converted into plant-available form (NO_3_^−^) through mineralization and nitrification processes. At the same time, ammonium is preferred by soil microorganisms, making it prone to immobilization^25,42^. The high pH values (8.0–8.8) (Fig. 1a,d) observed in soil amended with BSFFF could have triggered gaseous losses through ammonia volatilization and reduced the nitrification process as indicated by the higher ratios of ammonium to nitrate^43^. The pH values above 7.5 favour nitrogen loss through ammonia volatilization^44^ because an increase in pH has been shown to increase the dissociation of ammonium to ammonia gas, thus shifting the equilibrium to ammonia, which eventually evaporates^45^.

**Table 5:** Selected physical and chemical characteristics of the experimental soil, and organic fertilizers.

Parameter	pH (1:2.5 water)	Bulk density	Mineral N	Total N	TOC	SOM	Available P (ppm)	Exchangeable cations (cmol kg^−1^)			% sand	% clay	% silt	Textural class
		(g cm^−3^)	(mg kg^−1^)	(%)				K	Ca	Mg				
**Experimental soil**														
Test value	5.9	1.35	1.81	0.04	1.3	2.3	9.7	2.07	0.91	0.07	63	20.3	16.7	Sandy loam

Parameter	Moisture (%)	pH	EC (mS cm^−1^)	Ammonium	Nitrate	TOC	Total N	Total P	Total K	Total Ca	Total Mg	C/N ratio		
				(mg kg^−1^)		(%)								
**Organic fertilizers**														
BSFFF	30.1	7.7	2.7	74.4	1.39	35.2	2.1	1.16	0.17	0.19	0.16	16.8		
SAFI	29.8	6.4	6.1	39.4	92.3	45.1	3.0	1.23	1.49	0.29	0.43	15.0		

*TOC* total organic carbon, *SOM* soil organic matter, *BSFFF* black soldier fly frass fertilizer, *SAFI* commercial organic fertilizer, n = 3.

The higher soil nitrate concentration (Table 1) and nitrification rates (Table 2) realized using SAFI could be partly attributed to initially low ammonium/nitrate ratio (Table 1) and lower soil pH values (< 7.0) during experiments (Fig. 1a,d). This agrees with previous studies that reported higher autotrophic nitrification under conditions of low ammonium and low pH^46^. Therefore, the low nitrification rates observed in BSFFF amended soil could also be attributed to the toxic effects of high ammonium and alkaline conditions on the enzymatic activities of nitrifying bacteria^47^. Although, the current studies found slightly higher populations of bacteria (Fig. 1b,e) and fungi (Fig. 1c,f) in BSFFF treated soil than SAFI treatments, the strains of nitrifiers present, their functional roles, and influence on enzymatic activities were not determined. Future studies would be necessary to investigate the effect of the BSFFF amendment on the abundance and diversity of nitrifying bacteria, and enzymatic activities, to generate accurate conclusions on the nitrification process.

Conversely, the net N immobilization associated with SAFI could be attributed to the lignin and polyphenols present in the biochar used to make SAFI^31,48,49^. The recalcitrant carbon in biochar is resistant to microbial decomposition and consequently causes delays in N mineralization^50,51^. The low total N concentration, such as that observed in soil amended with SAFI (Fig. 2a,d), has also been found to stimulate immobilization and slow down N release^40^. Our sister paper^21^ suggested supplementation with mineral N fertilizers to compensate for N immobilization and improve synchrony of N supply for optimal crop production. This agrees with Kaleem Abbasi and Khaliq^34^, who reported accelerated mineralization and nitrification rates in soils amended with a combination of mineral N and organic fertilizers. However, the higher and net positive values of nitrification and nitrification rates observed during the long rains (Table 2) suggest that continued amendment of soil using BSFFF could gradually increase the nitrification rate and mineral N release for plant uptake.

**Figure 3:**
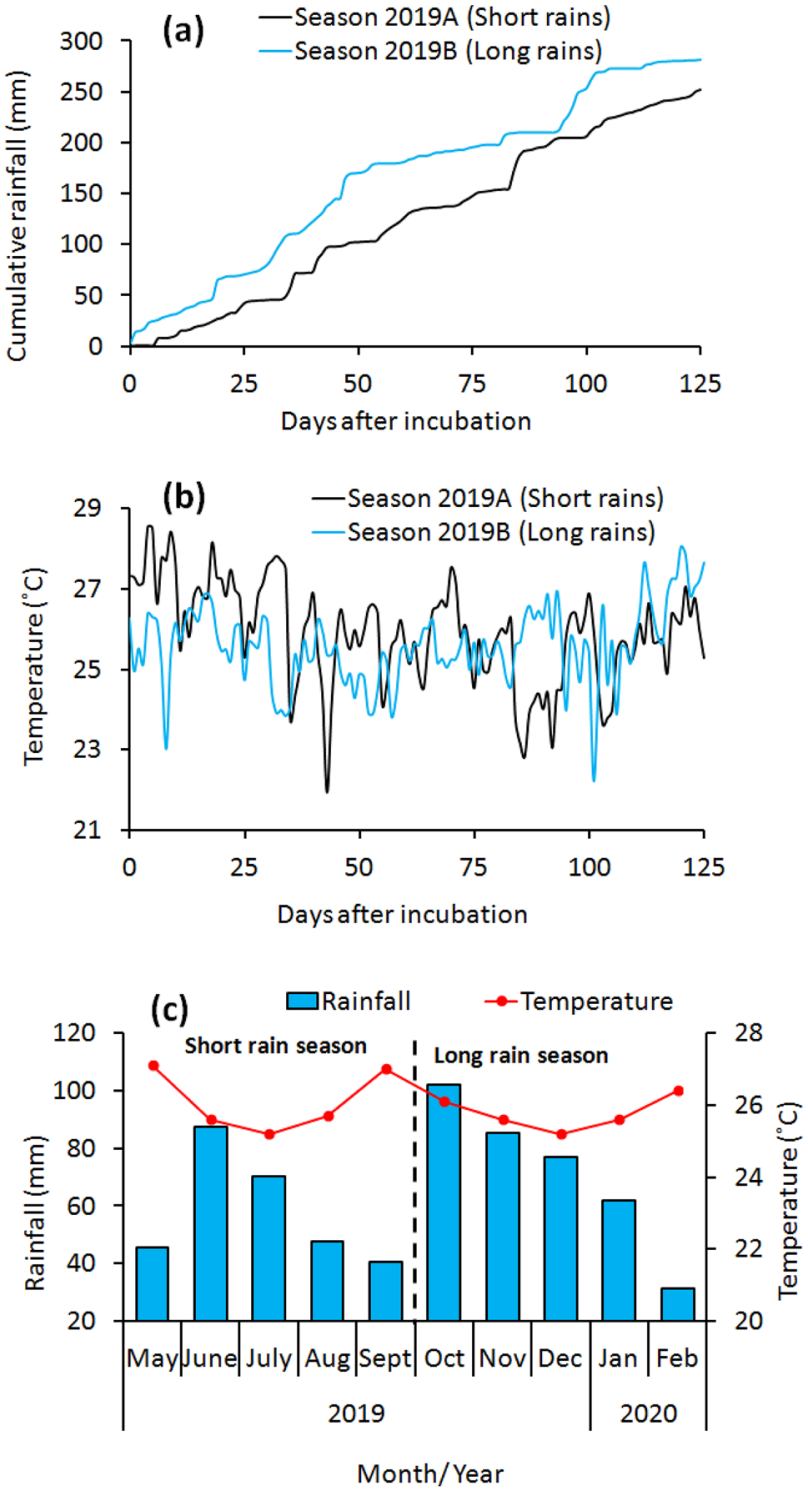
Cumulative rainfall (**a**), daily temperature (**b**) and monthly rainfall and temperature (**c**) during experiments.

The current study revealed higher nutrients concentrations (Fig. 3 and Table 3). Nutrient release (Table 4) in fertilizer amended soils compared to unfertilized soil is consistent with previous studies that reported the resultant effect of low soil fertility in Kenya^3,52,53^ and most countries in SSA^1,2^. It recommended regular fertilizer inputs for soil fertility improvement^23,54–56^. Therefore, the application of high-quality fertilizers (such as the BSFFF) can improve soil health and crop productivity^20,21^ by increasing the availability of nutrients (such as nitrogen and phosphorus), which are the most limiting nutrients to crop production in SSA^2^.

The amount of N released using BSFFF was higher than that achieved using farmyard manure and tithonia in the soils of central Kenya^57^. The higher concentrations and nutrients release capacity associated with BSFFF could be attributed to the good quality of the fertilizer and its high mineralization rate, as previously reported^27^. The increased phosphorus availability achieved in soil treated with BSFFF could be partly attributed to the high soil pH values (8.0–8.9) (Fig. 1a,d) that do not favour P fixation, which is common in the acidic soils of Kenya^52,53^ and SSA^2^. Acidic soils (< pH 6) have high iron and aluminium oxides that react with phosphate ions to form insoluble compounds, thus reducing the quantity of P available for plant growth^58,59^. Findings from the current study concur with those of von Arb et al.^60^, who reported an increase of available soil P in organic farming systems of Central Kenya. Therefore, this study highlights the role of the BSFFF amendment in ameliorating P availability by causing a liming effect in soils with low pH.

The high concentration and release of potassium observed in SAFI amended soil could be attributed to the initially high K concentration in SAFI (Table 5). This is in agreement with previous studies that reported high potassium concentration in biochar-fortified organic fertilizers^48,61^. Nevertheless, the quantities of potassium released by soil amended with BSFFF were sufficient to produce most cereals, legumes, and vegetable crops.

The increased release of N, P and K observed during the long rainy season (Table 4) indicates the role of soil moisture in nutrients release from organic fertilizer. With continued application, BSFFF could be solely relied on for providing nutrients for crop production. This would save farmers from the expensive mineral fertilizer that is less effective in soils with multiple degradation challenges^4,23^, and cater for organic farmers who exclusively rely on organic fertilizers for crop production. Our sister paper supported this assertation, which reported higher growth, yield and nutrient uptake associated with maize grown using BSFFF compared to commercial organic (SAFI) and mineral (urea) fertilizers^20^.

The higher populations of soil bacteria (Fig. 1b,e) and fungi (Fig. 1c,f) achieved using BSFFF indicate that in addition to increasing nutrient supply, this frass fertilizer has the potential to increase soil microbial abundance and diversity, which is key for improving biological soil fertility. The current findings agree with previous studies that have reported the additional benefits of using insect frass fertilizer (such as increased microbial abundance and activity, deeper plant root growth and suppression of plant pathogens)^9,11,12,22^. Furthermore, the BSFFF takes a shorter time to generate (5 weeks) than conventional composts (8–24 weeks)^18^. Therefore, findings from this study are crucial in changing attitudes towards organic fertilizer use, with the advantages of higher nutrient release capacity, less bulkiness and shorter production time associated with BSFFF.

## Conclusion

The current study has demonstrated that BSFFF has a high potential to supply adequate nutrients for optimal crop production. The higher ammonification and low nitrification rates indicate that nitrogen immobilization could occur at the initial stages of crop growth. Therefore, mineral N supplementation would be necessary to compensate for net immobilization and improve synchrony of N release for plant growth. Nevertheless, the increase in nutrient release and nitrification rates during the long rainy season implies that the continued application of BSFFF would increase N mineralization and nutrient release. The significantly higher amounts of P released by soil amended with BSFFF indicate its key role in enhancing P availability through its liming effect.

Furthermore, the higher population of soil bacteria and fungi associated with BSFFF amended soil underline its potential for improving biological soil fertility. Therefore, BSFFF is recommended for a sustainable enhancement of soil health and crop productivity, thus reducing overdependence on low-quality organic fertilizers and expensive mineral fertilizers. Future studies should determine mid- and long-term patterns in nutrient release, abundance, diversity and functional roles of bacteria and fungi in soils amended with BSFFF.

## Methods

### Description of the study site

Field experiments were set up for two seasons (April–September 2019 and October 2019–March 2020) at the Kenyatta University Teaching and Demonstration farm (1°10′59″ S, 36°55′34″ E, 1580 m above sea level), Nairobi County, Kenya. Nairobi County receives bimodal rainfall with annual averages of 925 mm and mean monthly temperatures of 21–28 °C (www. meteo.go.ke). The first rainfall season (short rains) starts from March to June, while the second season (long rains) runs from October to January. Cumulative rainfall totals of 252 and 281 mm were received during the short and long rainy seasons experiments, respectively (Fig. 3a). Mean monthly rainfall of 40 and 87 mm, and 31–102 mm were received during the short and long rainy seasons experiments, respectively (Fig. 3c). Mean daily temperatures of 22–29 °C, and 22–28 °C were recorded during the short and long rainy seasons, respectively (Fig. 3b). The short rainy season had higher mean monthly temperatures than the long rainy season (Fig. 3c).

Soils in the study site are Acric Ferralsols^62^ characterized by low organic matter, shallow depths, and low pH levels. Before the onset of experiments, soils were sampled (0–20 cm) for determination of total organic N, total organic carbon, available phosphorus (P), exchangeable cations [potassium (K), calcium (Ca), and magnesium (Mg)], pH, and soil texture following procedures described by Okalebo et al.^63^. Table 5 presents the characteristics of the experimental soil.

### Source of organic fertilizers

The experiment involved two organic fertilizers: the black soldier fly frass fertilizer (BSFFF) and commercial organic fertilizer (SAFI). The BSFFF was a product obtained from the feeding of BSF larvae on brewery spent grain (sourced from Kenya Breweries Limited, Nairobi) at the Animal Rearing and Quarantine Unit of the International Centre of Insect Physiology and Ecology (*icipe*), Nairobi. The BSF larvae were reared in metallic trays using a rearing substrate hydrated to approximately 70 ± 1% moisture content, following procedures described by Beesigamukama et al.^18^. The frass obtained was composted inside a greenhouse using the heap method. During composting, frass heaps of 1 m height and 4 m long were built on surfaces lined with polythene sheets and hydrated to 55–65% moisture content. After 5 weeks, a mature and stable frass product was obtained and used in the experiments as BSFFF. Details of the entire composting process up to the compost maturity stage are described in our sister paper^21^.

The SAFI was sourced from Safi Organics Limited (http://safiorganics.co.ke/) located in Mwea town, Kirinyaga County, Kenya. It was a mixture of composted chicken manure, biochar, and rock phosphate. Table 5 presents selected physical–chemical characteristics of the organic fertilizers used in the experiments.

### Experimental set up

This study was carried out alongside agronomic experiments aimed at determining the nitrogen fertilizer equivalence value of BSFFF and synchrony of N release for maize production^21^. The BSFFF and SAFI were applied at rates of 0 and 5 t ha^−1^, according to an organic fertilizer rate that had been previously used in central Kenya^31,57,64^. Thus, parallel experiments were carried out for a period equivalent to two maize cropping seasons (April–September 2019 and October 2019–March 2020) to investigate the ammonification and nitrification processes, rates of N mineralization and nitrification, and the quantities of nutrients released by soil amended with BSFFF and SAFI.

### Soil sampling and incubation

From each plot, the soil was collected from 0 to 20 cm depth before applying organic fertilizers. The soil was manually sorted to remove objects, stones, and clods bigger than 2 mm. The soil was then homogenized by hand mixing in a basin. The two organic fertilizers were separately mixed with the soil at the rate of 5 t ha^−1^. The moisture content of the mixture (soil-organic fertilizer) was adjusted to 60% soil water holding capacity using distilled water. Two hundred grams of the mixture were placed in an air-permeable ziplock bag sealed to prevent water entry and nutrient loss^36,40^. The ziplock bags were buried at 10–20 cm depth in respective plots in the field. At the same time, 200 g of unamended soil from each plot (as control) were placed in ziplock bags and buried at the same depths (10–20 cm) in each of the respective plots.

Five bags were randomly buried per replicate, giving a total of 15 bags per treatment at the beginning of each cropping season. The bags were retrieved at 15, 30, 70, 91, and 125 days of incubation corresponding to seedling, vegetative, tasseling, silking, and harvesting stages of the maize crop growth. The positions of the bags were marked using pegs to avoid disturbance during weeding and for easy retrieval. The retrieved bags were labelled on each sampling date, placed in airtight polythene bags, and carried in a cool box containing ice blocks to reduce microbial activities during transportation. The samples were used to determine soil mineral N (ammonium and nitrate) concentration, pH, fungi and bacterial populations, and other nutrient concentrations.

### Nitrogen mineralization

The concentrations of nitrate (NO_3_^–1^) and ammonium (NH_4_^+^) in unamended soil and soil amended with organic fertilizers were used to calculate N mineralization and nitrification rates at each sampling time using Eqs. (1) and (2)^40^. The nitrification ratio was calculated by dividing the concertation of ammonium (mg kg^−1^) at each sampling period by concentrating nitrate (mg kg^−1^) during the same period.

1 $$Nitrogen\, mineralization\, rate \left(mg\, N\, {kg}^{-1}{day}^{-1}\right)=\frac{\left[{{NO}_{3}^{-}+{NH}_{4}^{+}\left(mg\, {kg}^{-1}\right) t}_{i+k}-{{NO}_{3}^{-}+{NH}_{4}^{+}\left(mg\, {kg}^{-1}\right) t}_{i}\right]}{{t}_{i+k}-{t}_{i}}$$

2 $$Nitrification\, rate \left(mg\, N\, {kg}^{-1}{day}^{-1}\right)=\frac{\left[{{NO}_{3}^{-}\left(mg\, {kg}^{-1}\right) t}_{i+k}-{{NO}_{3}^{-}\left(mg\, {kg}^{-1}\right) t}_{i}\right]}{{t}_{i+k}-{t}_{i}}$$

where, *t*_*i*_ represents sampling times *i* = 0, 1, 2, 3, … t_i + k_ is *t*_*i*_ plus time *k* intervals where *k* = 1, 2, 3, 4,…

### Nutrient released by unamended soil and soils amended with organic fertilizers

The concentrations of nutrients (N, P, K, Ca, and Mg) released by unamended soil and soil amended with organic fertilizers at the maturity stage (125th day of incubation) of each cropping season were used to calculate the cumulative quantities of nutrients released throughout the maize growing season. The amounts of nutrients (N, P, K, Ca, and Mg) released were expressed on a kg per hectare basis using Eq. (3). The amount of N released was computed using the mineral N (ammonium and nitrate) data.

3 $$Nutrient\, released \left(kg\, {ha}^{-1}\right)=\frac{nutrient\, concentration \left({mg\, kg}^{-1}\right)\times mass\, of\, soil\, layer \left(kg\right)}{{1,000,000}}$$

where, $$mass\, of\, soil\, layer \left(kg\right)=soil\, bulk\, density \left(kg {m}^{-3}\right)\times volume\, of\, soil\, layer \left({m}^{3}\right)$$

$$volume \,of\, soil\, layer \left({m}^{3}\right)=area\, of\, one\, hectare \left({m}^{2}\right)\times depth\, of\, soil\, layer (m)$$

### Laboratory analysis methods

The pH and electrical conductivity (EC) were determined using extracts of 1:10 and 1:2.5 (w/v) for organic fertilizer to distilled water and soil to distilled water, respectively. The contents were then shaken for 1 h, 180 revolutions min^−1^, on an orbital and linear shaker (MI0103002, Foure’s Scientific, Guangdong, China). Then, the pH and EC were read directly using a pH (AD1000, Adwa, Bucharest, Romania) and EC meter (AVI, Labtech, Mumbai, India), respectively Okalebo et al.^63^.

The mineral N (nitrate and ammonium) was extracted from organic fertilizers and soil using 0.5 M potassium sulphate at a ratio of 1:10 (w/v). The nitrate and ammonium concentrations in solutions after filtration were determined by colourimetric methods at 419 and 655 nm, respectively, as described by Okalebo et al.^63^. The populations of soil bacteria and fungi were determined by culturing using nutrient agar for bacteria and potato dextrose agar for fungi. The number of colony-forming units (CFU) per treatment were counted after 24–48 h, and data were expressed as CFU g^−1^. Total organic carbon of organic fertilizers and soil was determined using the wet oxidation method^65^.

The total N, P, K, Ca, and Mg of organic fertilizers were extracted using the acid digestion method^63^. From this extract, total N, P, and K were determined using the Kjeldahl digestion and distillation method^66^, UV–Vis spectrometry^63^, and flame photometry^63^, respectively. The total Ca and Mg concentrations were determined using atomic absorption spectrometry (AAS)^63^ at 422.7 and 285.2 nm, respectively (iCE 3300 AA system, Thermo Scientific, Shanghai, China). Available P and exchangeable Ca and Mg in soil were determined using Bray 2^63^ and AAS, respectively, while exchangeable K was determined using flame photometry. Total N in soil was determined using Kjeldahl digestion and distillation method^66^, while soil texture and bulk density determined using the Bouyoucos and core sampling methods, respectively^63^.

### Data analysis

Before statistical analysis, data were tested for normality using the Shapiro–Wilk test. Analysis of variance tests was performed on data on soil pH and concentrations of ammonium, nitrate, nutrients (N, P, K, Ca, Mg), fungi, and bacteria using a linear mixed-effect model with “lmer” function from the package “lme4”. Fertilizer treatments and incubation time were kept as fixed effects, whereas replication was a random effect. Data on amounts of nutrients were analyzed using a one-way analysis of variance test. Computation of least squares means was done using “lsmeans” package, followed by mean separation using adjusted Tukey’s method implemented using “cld” function from the “multicompView” package. Data were analyzed separately for each season. All the statistical analyses were conducted using R software version 3.6.0^67^.

## Data Availability

All relevant data are presented in the paper.
